# Frailty in middle age is associated with frailty status and race-specific changes to the transcriptome

**DOI:** 10.18632/aging.102135

**Published:** 2019-08-08

**Authors:** Calais S. Prince, Nicole Noren Hooten, Nicolle A. Mode, Yongqing Zhang, Ngozi Ejiogu, Kevin G. Becker, Alan B. Zonderman, Michele K. Evans

**Affiliations:** 1 Laboratory of Epidemiology and Population Sciences, National Institute on Aging, National Institutes of Health, Baltimore, MD 21224, USA; 2 Laboratory of Genetics and Genomics, National Institute on Aging, National Institutes of Health, Baltimore, MD 21224, USA

**Keywords:** frailty, race, gene expression, sequencing, health disparities, middle age

## Abstract

Frailty is an aging-associated syndrome resulting from diminished capacity to respond to stressors and is a significant risk factor for disability and mortality. Although frailty is usually studied in old age, it is present in mid-life. Given the increases in mortality statistics among middle-aged Americans, understanding molecular drivers of frailty in a younger, diverse cohort may facilitate identifying pathways for early intervention. We analyzed frailty-associated, genome-wide transcriptional changes in middle-aged blacks and whites. Next generation RNA sequencing was completed using total RNA from peripheral blood mononuclear cells (n = 16). We analyzed differential gene expression patterns and completed a parametric analysis of gene set enrichment (PAGE). Differential gene expression was validated using RT-qPCR (n = 52). We identified 5,082 genes differentially expressed with frailty. Frailty altered gene expression patterns and biological pathways differently in blacks and whites, including pathways related to inflammation and immunity. The validation study showed a significant two-way interaction between frailty, race, and expression of the cytokine *IL1B* and the transcription factor *EGR1.* The glucose transporter, *SLC2A6*, the neutrophil receptor, *FCGR3B,* and the accessory protein, *C17orf56,* were decreased with frailty. These results suggest that there may be demographic dependent, divergent biological pathways underlying frailty in middle-aged adults.

## Introduction

Frailty is a clinical syndrome that has been defined by several different assessments as a loss of physiological reserve, thus hindering recovery from endogenous and exogenous stressors [1–3]. Frail individuals have increased risk for several adverse outcomes including: falls, disability, complications following surgery, and premature mortality [4–7]. The most recent data finds that the overall prevalence of frailty in adults 65 years old and older is approximately 15% [8]. In the United States, the prevalence of frailty in the elderly is highest in women, racial/ethnic minorities, and individuals at lower incomes [8–10].

While frailty has been primarily examined as a clinical entity of the aged, frailty can occur in adults 45 – 64 years old. We have previously shown in a longitudinal cohort study of blacks and whites in Baltimore that frailty prevalence among these individuals is approximately 7% in adults 35-44 years old and over 13% in whites between the ages of 45-54 years old [10]. Other studies in young to middle-aged diverse cohorts, show that frailty, grip strength, and low appendicular skeletal muscle mass are associated with poor health outcomes [10–12]. Frailty, because of its link to risk for mortality, is an increasingly important entity to study in younger cohorts in light of the increase mid-life mortality among Americans across racial and ethnic groups [13]. With an expected increase in the US 45 – 64 year old population by 15% by 2060, it is imperative to identify functional biological markers of frailty to identify potential interventions that could aid in ameliorating the phenotype.

Identifying a single operational definition for frailty in a population has, thus far, resulted in close to 30 different assessments [14]. The Cardiovascular Health Study (CHS)/Fried Index [3] is a popular and well-validated assessment that characterizes frailty by phenotype based on the presence of: shrinking (unintentional weight loss of > 10 pounds by direct measurement of weight), weakness, poor endurance and energy (self reported), slowness (time to walk 15 feet), and low physical activity (weighted score of kilocalories expended per week). Although seminal to the field of frailty research, diagnosing frailty using the CHS frail phenotype requires an in person physical with techniques that are not typically available to primary care physicians. The International Academy on Nutrition and Aging FRAIL scale is an easily administered questionnaire [9] that has been validated in community based, racially diverse populations consisting of middle-aged adults [9,10,15]. Most clinical and molecular studies assessing frailty utilizing the CHS index and have focused on older, largely white cohorts.

Complementary to diagnosing frailty based on phenotypic changes is to identify biomarkers for frailty. Potential dysregulated pathways associated with frailty include: inflammation, apoptosis, calcium homeostasis, neuronal signaling (central nervous system and peripheral nervous system), hormone regulation, and gene expression/transcriptional regulation [16]. Although characterized as a chronic state of inflammation [17], circulating cytokine concentrations vary amongst frail populations which is a reflection of the complexity of the syndrome that could be attributed to genetic variability and participant demographics [17]. Thus, examining gene expression profiles can serve as a promising molecular pathway to investigate frailty. The Vitality 90+ study [18], a longitudinal study that focuses on longevity in the oldest old, is the largest study to date to examine frailty-associated changes to the transcriptome of nonagenarians. However, the focus of this study was to examine transcriptional changes in the context of survival and mortality and was not necessarily focused on frailty-associated changes [18].

Previous studies do not examine whether there are differences in the underlying biological processes associated with the frail phenotype in the context of race and younger age groups. In this study, we have chosen to examine frailty-associated, genome-wide transcriptional changes in frail and non-frail middle-aged blacks and whites. This will permit us to either confirm that racially diverse middle-aged individuals with frailty have similar gene expression profiles as older European ancestry adults from previously published work [18] or to identify novel gene expression profiles that may be specific for younger or non-European ancestry individuals. We hypothesize that gene expression is influenced by frailty status and demographic characteristics including race.

## RESULTS

### Frailty-associated global changes to gene expression

**Figure 1:**
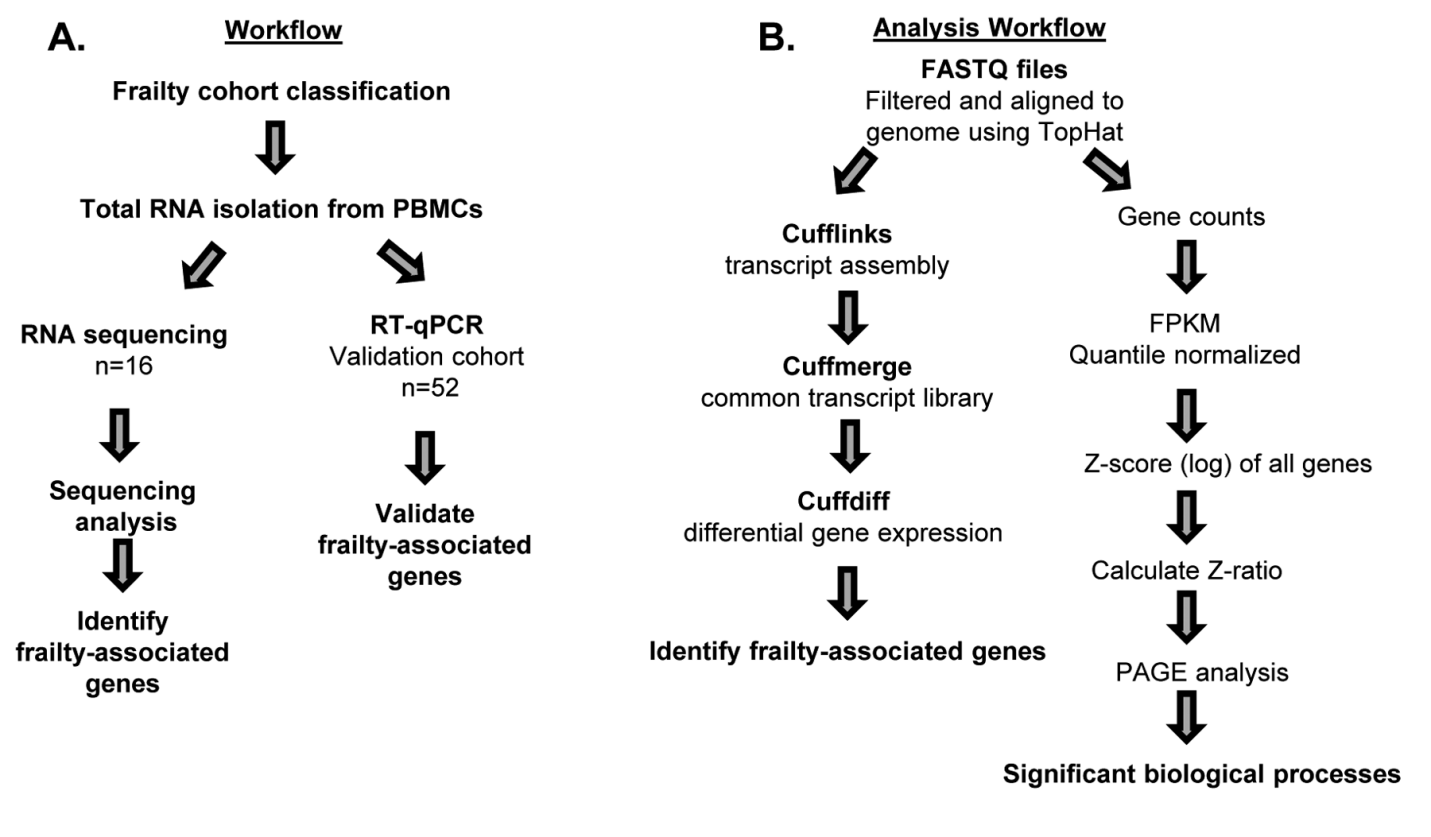
**Workflow and schematic representation of RNA sequencing and RT-qPCR analysis.** Overview of study design for RNA sequencing (n=16) and RT-qPCR in validation cohort (n=52) (**A**), and RNA sequencing analysis for alignment and identification of frailty-associated genes (**B**).

The overall study design and RNA sequencing and analysis workflow are shown in Figure 1. We classified frailty in blacks and whites in the RNA sequencing and validation cohorts (Figure 1A) utilizing the FRAIL scale. To identify genome-wide transcriptional changes associated with frailty in middle-aged adults, we isolated total RNA from peripheral blood mononuclear cells (PBMCs) from 16 non-frail, pre-frail/frail blacks and whites between the ages of 45-49 years old. (Table 1A). We performed next generation RNA sequencing and used TopHat to align the sequences to human genome NCBI build Version 38 (Figure 1B). The percentage of mapped reads ranged between 66-92% and the total mapped reads ranged between 14 and 44 million. RNA-seq data was analyzed as shown in Figure 1B.

**Table 1:** Demographic information for frailty cohorts.

**A**	** RNA sequencing cohort**			
	**Non-frail**	**Pre-frail/Frail**		
n	8	8		
Age (mean (sd))	48.09 (1.21)	47.85 (1.84)		
Sex = Women (%)	4 (50.0)	4 (50.0)		
Race = White (%)	4 (50.0)	4 (50.0)		
PovStat = Below (%)	2 (25.0)	4 (50.0)		
				
**B**	** Validation cohort**			
**Validation cohort**				
	B:Non-frail	W:Non-frail	B:Frail	W:Frail
n	13	13	13	13
Age (mean (sd))	47.51 (1.59)	47.23 (1.26)	47.04 (1.62)	47.58 (1.80)
Sex = Women (%)	7 (53.8)	6 (46.2)	7 (53.8)	6 (46.2)
PovStat = Below (%)	6 (46.2)	3 (23.1)	8 (61.5)	5 (38.5)

**Figure 2:**
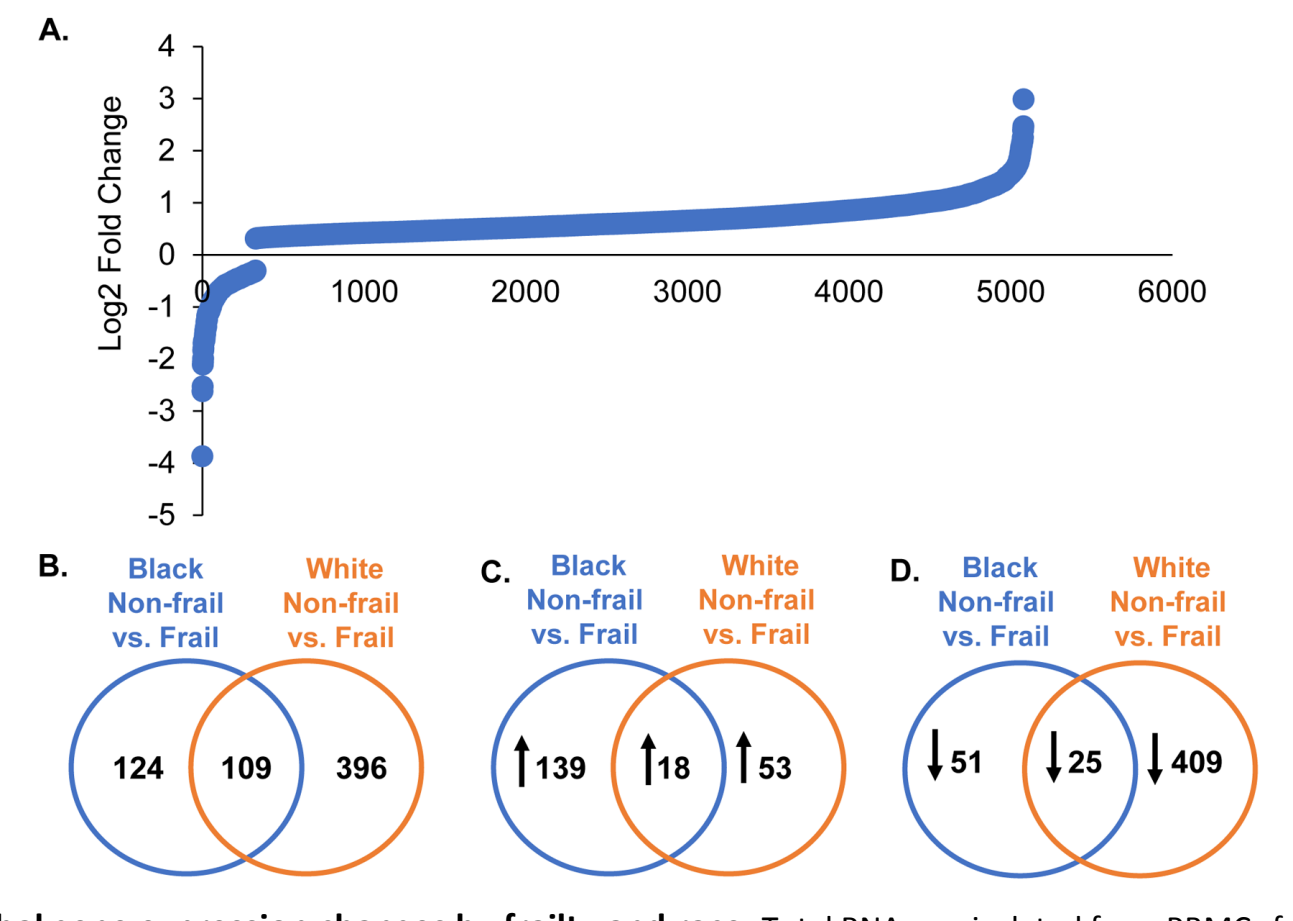
**Global gene expression changes by frailty and race.** Total RNA was isolated from PBMCs from non-frail and frail blacks and whites (n=16). Differential gene expression was assessed using RNA sequencing. The graph shows log2 fold change of genes significantly altered with frailty (5,082 genes) (**A**). A list of all these genes can be found in Supplementary Table 1. Venn diagrams of the total number of significant differentially expressed genes in blacks and whites with frailty (**B**). Significantly increased (**C**; up arrow) and decreased (**D**; down arrow) genes in blacks and whites with frailty.

Frailty was associated with differential expression of 5,028 genes (Figure 2A; Supplementary Table 1). When stratifying by race, as displayed in the Venn diagrams, 124 genes in blacks, 396 in whites, and 109 overlapping genes between blacks and whites were significantly and differentially expressed in the context of frailty (Figure 2B). Genes significantly different between non-frail and frail blacks and whites are listed in Supplementary Table 2 and Supplementary Table 3, respectively. When comparing the number of genes that were higher in abundance in frail individuals, we identified 139 genes in blacks, 53 genes in whites, and 18 overlapping genes between blacks and whites (Figure 2C). When comparing the number of genes with lower abundance in frail individuals, we identified 51 genes in blacks, 409 genes in whites, and 25 overlapping in blacks and whites (Figure 2D).

**Figure 3:**
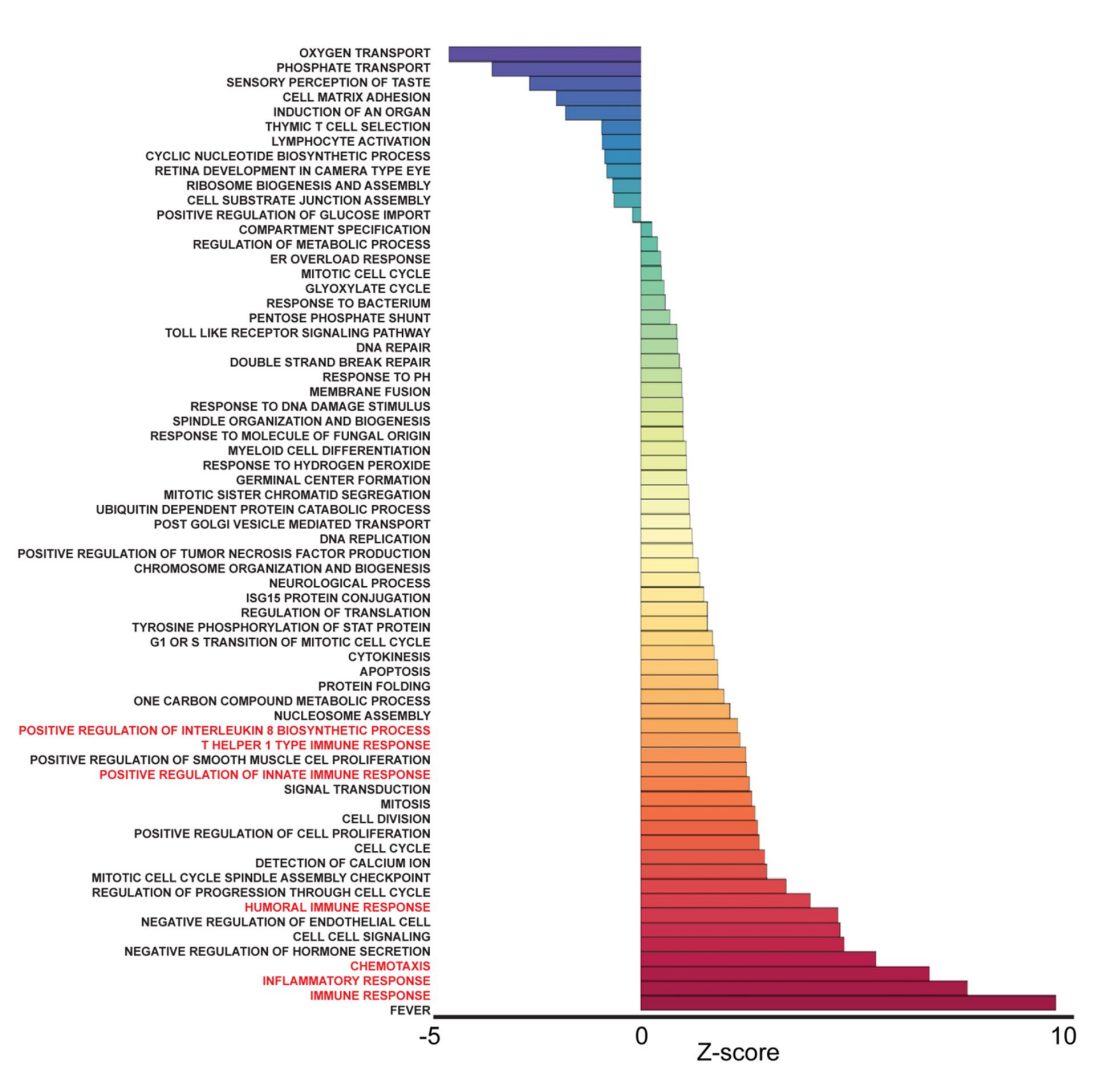
**Frailty-associated biological pathways in blacks.** Differentially expressed genes between non-frail and frail blacks were imputed into Parametric Analysis of Gene Set Enrichment (PAGE) analysis. Significantly changed gene sets associated with biological processes, organized by Z-score, are shown here. GO terms associated with inflammation and immune response are highlighted in red.

**Figure 4:**
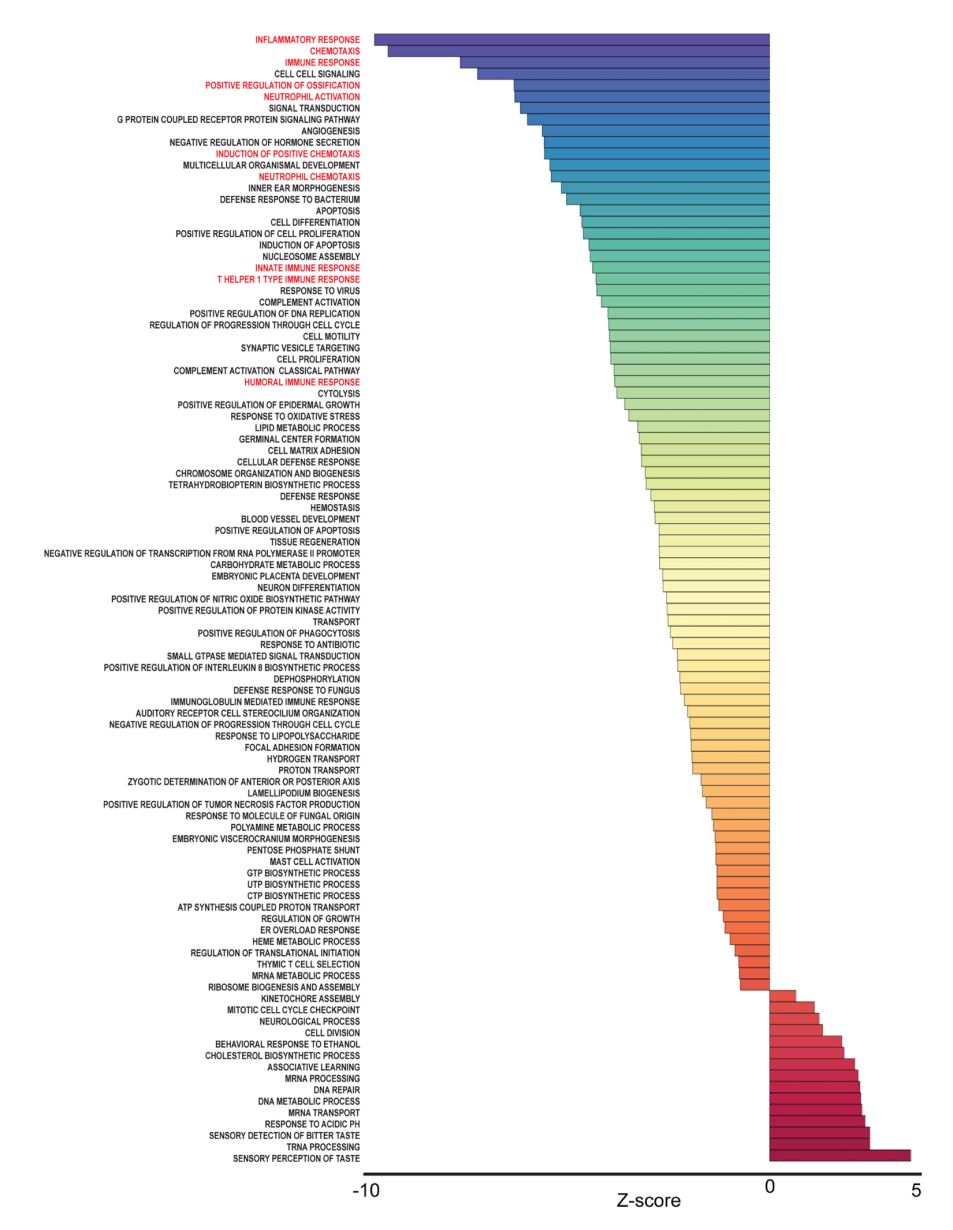
**Frailty-associated biological pathways in whites.** Differentially expressed genes between non-frail and frail whites were imputed into Parametric Analysis of Gene Set Enrichment (PAGE) analysis. Significantly changed gene sets, associated with biological processes, organized by Z-score, are shown here. GO terms associated with inflammation and immune response are highlighted in red.

To identify pathways associated with frailty in blacks and whites, we used the quantile normalized Fragments Per Kilobase of transcript per Million mapped reads (FPKM) to calculate Z-scores [19,20] (Figure 1B). The Z-scores were transformed (natural logarithm) and used to calculate Z-ratios that were imputed into PAGE analysis to identify relevant biological processes. We identified enriched gene ontology (GO) sets associated with biological processes comparing non-frail and frail blacks (Figure 3) and whites (Figure 4). Importantly, there were fewer pathways identified in blacks when compared to whites. The most enriched pathways in frail blacks were fever, immune response, inflammatory response, chemotaxis, and negative regulation of hormone secretion. The most downregulated pathways in frail blacks were oxygen transport, phosphate transport, sensory perception of taste, cell matrix adhesion, and induction of an organ. Conversely, in frail whites, the most enriched pathways were sensory perception of taste, tRNA processing, detection of chemical stimulus involved in sensory perception of bitter taste, response to acidic pH, and mRNA transport. The most downregulated pathways in frail whites were inflammatory response, chemotaxis, immune response, cell-cell signaling, and positive regulation of ossification. We highlighted GO terms in red that were associated with inflammation, as the pathway Z-scores for frailty varied greatly between blacks and whites (Table 2). This suggests that inflammatory pathways could be a driver for racial differences in frailty. There were 19 overlapping pathways comparing blacks to whites, however most of the pathway Z-scores were in opposite directions, only 2 were in the same direction: chemotaxis and cell matrix adhesion (Table 2). The complete list of biological processes, stratified by race, can be found in Supplementary Table 4 and Supplementary Table 5.

**Table 2:** Frailty-associated gene ontology terms with overlapping relationships in blacks and whites.

**blacks**	**Gene Ontology Term**	**whites**
9.05	CHEMOKINE ACTIVITY	-6.70
7.11	IMMUNE RESPONSE	-5.73
6.29	INTERLEUKIN 1 RECEPTOR BINDING	-4.98
6.28	INFLAMMATORY RESPONSE	-7.32
6.25	CYTOKINE ACTIVITY	-6.47
5.12	CHEMOTAXIS	7.08
4.42	NEGATIVE REGULATION OF HORMONE SECRETION	-4.18
4.34	CELL CELL SIGNALING	-5.41
3.69	HUMORAL IMMUNE RESPONSE	-2.87
3.16	REGULATION OF PROGRESSION THROUGH CELL CYCLE	-2.98
2.54	POSITIVE REGULATION OF CELL PROLIFERATION	-3.46
2.36	SIGNAL TRANSDUCTION	-4.62
2.17	T HELPER 1 TYPE IMMUNE RESPONSE	-3.21
2.12	NUCLEOSOME	-3.04
1.94	NUCLEOSOME ASSEMBLY	-3.33
1.91	PROTEIN BINDING	-4.36
1.68	APOPTOSIS	-3.52
-1.84	CELL MATRIX ADHESION	-2.38
-2.42	SENSORY PERCEPTION OF TASTE	2.62

Gene ontology Z-scores for non-frail versus frail blacks or whites are indicated.

### Interaction network analysis

**Figure 5:**
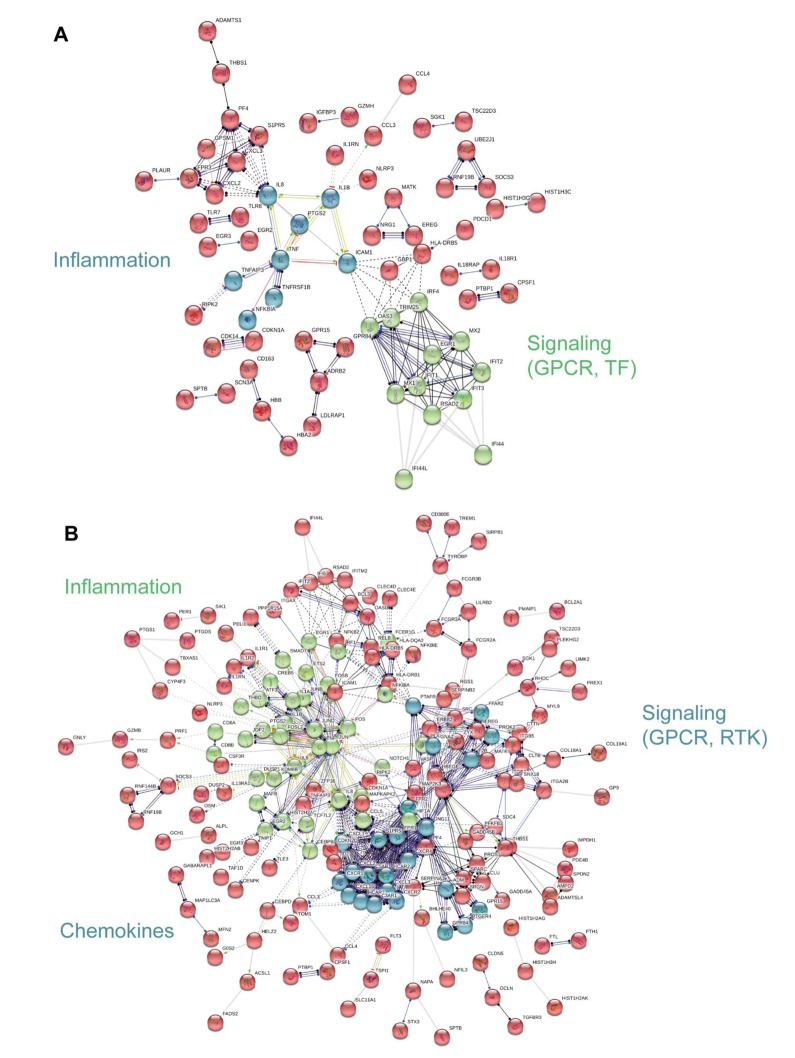
**Interaction network analysis of frailty-associated differential gene expression.** The interaction network for significant, differentially expressed protein coding genes with frailty in blacks (**A**) and whites (**B**) is shown. Network nodes represent proteins and lines (edges) represent protein-protein interactions. The solid lines represent direct interactions between proteins, dashed lines represent indirect interactions between proteins, grey lines represent putative protein interactions. The line colors represent the molecular action type: green (activation), dark blue (binding), black (reaction), red (inhibition), purple (catalysis), yellow (transcriptional regulation). The action effect is represented by the shape at the end of the line: arrow head (positive), perpendicular line (negative), dot (unspecified). The prominent functional clusters in blacks and whites are indicated.

To visualize functional connections between frailty-associated genes, we used protein-protein interaction analysis as a complement to the PAGE analysis [21]. The significant, differentially expressed, frailty-associated genes referenced in Figure 2B were imputed into the STRING database. In blacks, we identified 2 functional clusters, inflammation and signaling: G protein coupled receptor (GPCR) and transcription factor (TF) signaling (Figure 5A). In whites, we identified 3 functional clusters, inflammation, chemokines, and signaling: G protein coupled receptor (GPCR) and receptor tyrosine kinase (RTK) signaling (Figure 5B). The analysis yielded different, frailty-associated protein-protein interaction networks for blacks and whites.

### Gene expression changes associated with frailty and race

We designed a validation cohort to increase our sample size and to confirm our results using RT-qPCR. In our validation cohort (Table 1B), we increased our sample size to 52, including 14 participants from the RNA sequencing cohort. We selected genes based on the following criteria: pathways of interest, a significant log2 fold change of + 3, and the presence within functional clusters from network analysis (Figure 5). In addition, we chose genes from the Vitality 90+ study, which is a longitudinal study of successful aging in nonagenarians in Finland [18]. Previously, gene expression was examined in this cohort in the context of survival and mortality. Among the individuals examined were frail individuals. We reanalyzed this data set to identify frailty- associated genes and found 3 genes associated with frailty (*FAM116B*, *SLC2A6,* and *C17orf56*). Therefore, we included these genes in our data set.

**Figure 6:**
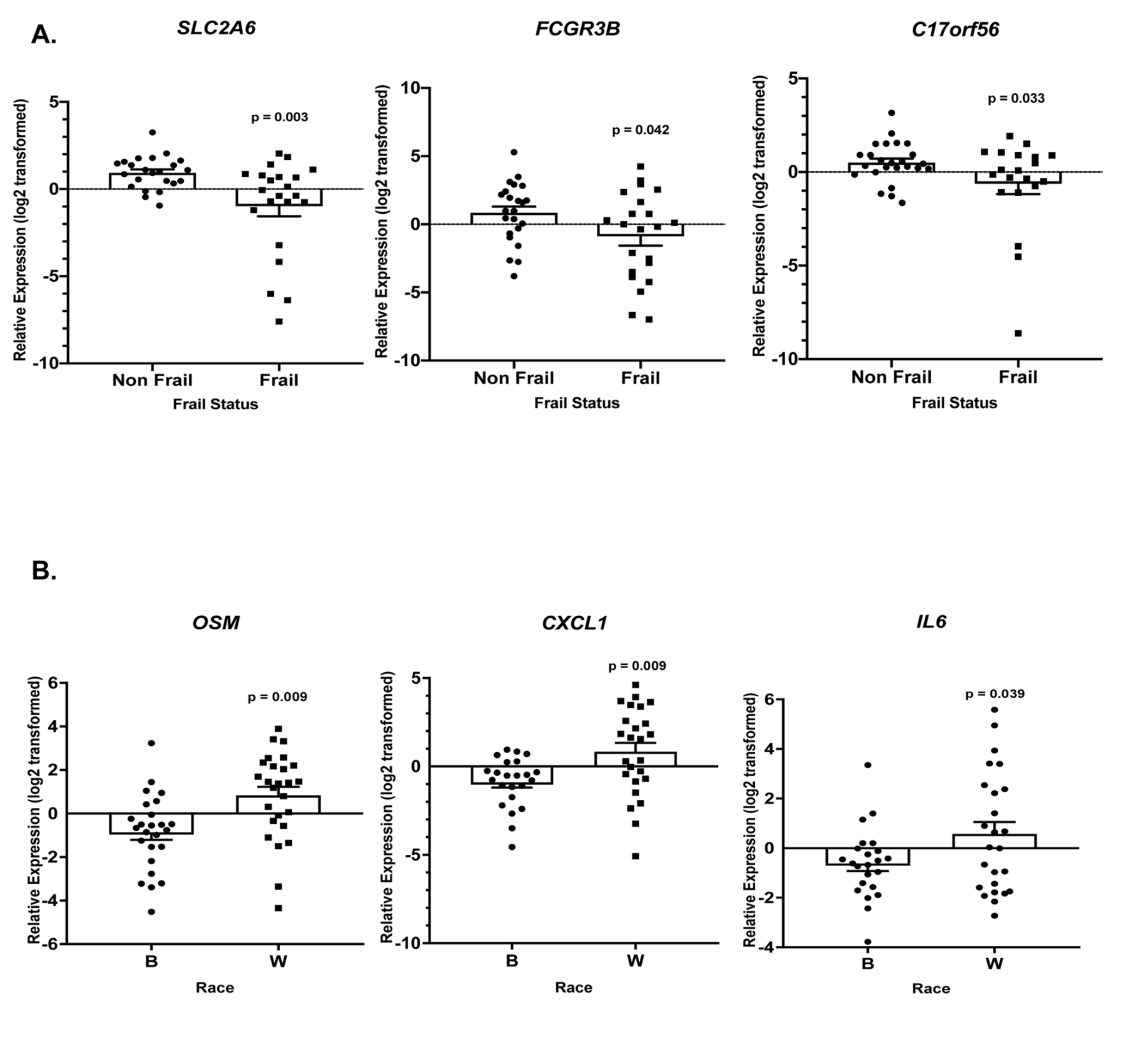
**Frailty and race-associated changes in gene expression in the validation cohort.** Total RNA was isolated from PBMCs from non-frail and frail blacks and whites in the validation cohort (Table 1B; n=52). Gene expression was analyzed using RT-qPCR with gene specific primers (Refer to Supplementary Table 6). The scatter plots show the relative expression (log2 transformed) in non-frail vs frail (**A**) and blacks (**B**) vs whites (W) in this same cohort (**B**). The open bars represent the mean and error bars show standard error of the mean. Significance was determined using linear regression models on the log2 transformed values.

**Figure 7:**
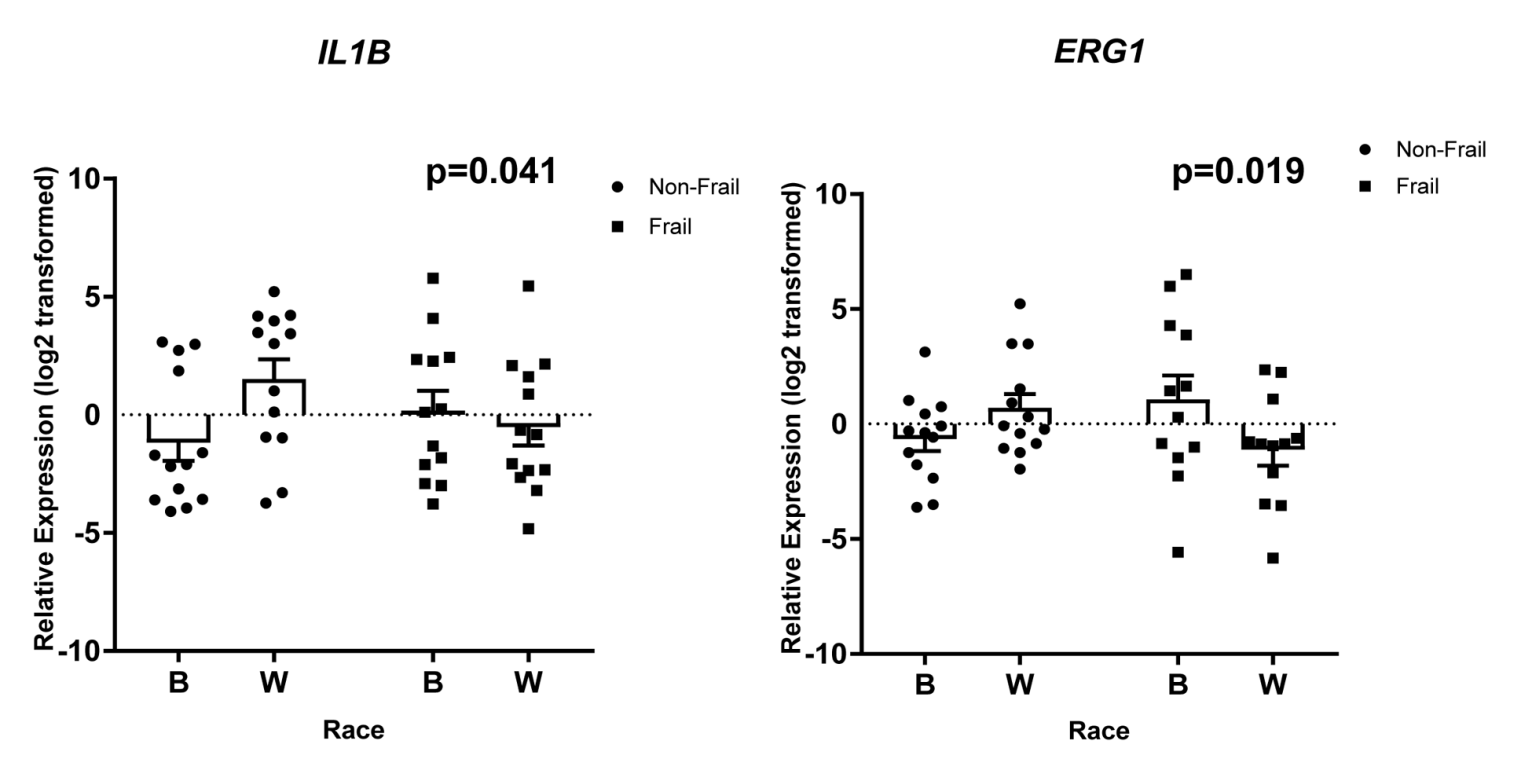
**Frailty-associated changes in gene expression with race.** Total RNA was isolated from PBMCs from non-frail and frail blacks and whites in the validation cohort (n=52). Gene expression was analyzed using RT-qPCR with gene specific primers. The scatter plots show the relative expression (log2 transformed) in non-frail and frail blacks (B) and whites (W). The open bars represent the mean and error bars show standard error of the mean. There is a significant two-way interaction between frailty status and race for *IL1B* (p=0.041) and *EGR1* (p=0.019). Significance was determined using linear regression models on the log2 transformed values.

Gene specific primers were designed and are listed in Supplementary Table 6. Of the 36 genes (*TNF*, *SLC2A6*, *RARA*, *OSM*, *LRCH3*, *IL15RA*, *IL6*, *IL1B*, *HBB*, *FCGR3B*, *FAM116B*, *CXCR1*, *CXCL1*, *CALCA*, *C17orf56*, *PF4*, *CXCL3*, *FPR3*, *CXCL2*, *PTGS2*, *ICAM1*, *GBP1*, *IRF4*, *TRIM25*, *OAS3*, *EGR1*, *IFIT1*, *IFIT3*, *RSAD2*, *IFI44*, *IFI44L*, *NOTCH1*, *CDKN1A*, *CCL5*, *MAPKAPK2*, *FPR2*) we observed statistically significant changes in the expression of 8 genes. Frailty was associated with significantly lower levels of solute carrier family 2, facilitated glucose transporter member 6 (*SLC2A6*), Fc fragment of IgG receptor IIIb (*FCGR3B*), and TEPSIN, adaptor related protein complex 4 accessory protein (*C17orf56*) (Figure 6A). Significantly higher levels of oncostatin M (*OSM*), C-X-C motif chemokine ligand 1 (*CXCL1*), and interleukin 6 (*IL6*) (Figure 6B) were identified in whites when compared to blacks, independent of frailty status which was not significantly associated with these genes. We also identified a significant two-way interaction between frailty and race for the expression of interleukin 1 beta (*IL1B*) and early growth response 1 (*EGR1*) (Figure 7). Sex was not a significant factor in any of the models.

### Correlation of *IL6* and *IL1B* gene expression with serum levels

To examine the relationship between *IL6* and *IL1B* gene expression and circulating cytokine levels, we measured serum IL6 and IL1β by ELISA. We did not identify a significant correlation between circulating cytokine levels and gene expression of *IL6* (r = -0.05; p = 0.76) or *IL1B* (r = -0.25; p = 0.08).

## DISCUSSION

In this present study, we have examined the transcriptome of non-frail versus frail middle-aged, community dwelling blacks and whites. Using RNA-seq, we identified over 5,000 differentially expressed genes associated with frailty. There were a larger number of genes differentially expressed in the context of frailty among whites when compared to blacks with a small number of genes that overlapped between the groups. However, there were more genes whose expression was increased among frail blacks when compared to frail whites. In contrast, there were more genes whose expression was decreased among frail whites when compared to frail blacks. Pathway analysis of these differentially expressed genes identified enriched gene ontology sets associated with biological processes; in blacks the most upregulated frailty associated biologic pathways were those associated with chemotaxis, inflammatory and immune response. In contrast, those same pathways were the most down-regulated among frail whites. While there were 19 overlapping pathways between blacks and whites, the directional pattern of these pathways was different. Using RT-qPCR, we validated expression patterns in genes that had a significant log2 fold change in expression, genes that were in functional clusters from our network analysis, genes that were in pathways of interest, or genes that we identified as frail associated and differentially expressed in the literature. Among the 36 genes studied, we identified 8 genes whose expression was influenced by frailty, race or the interaction of frailty and race. These included: solute carrier family 2, facilitated glucose transporter member 6 (*SLC2A6*), Fc fragment of IgG receptor IIIb (*FCGR3B*), and TEPSIN, adaptor related protein complex 4 accessory protein (*C17orf56*) oncostatin M (*OSM*), C-X-C motif chemokine ligand 1 (*CXCL1*), interleukin 6 (*IL6*), interleukin 1 beta (*IL1B*) and early growth response 1 (*EGR1*).

Previous work has been done to examine gene expression in frailty. Given that our cohort is middle-aged and diverse, it is noteworthy that we have identified such a large number of genes that are differentially expressed with frailty. This is also important in the context of other studies examining the transcriptome of frailty. Although there are few studies examining the transcriptome of frailty, as part of the Vitality study, gene expression was analyzed in the context of developing a mortality-predicting signature. Frailty status was included as a variable in the model, but gene expression was not analyzed based on frailty status. We reanalyzed these data to identify frailty-associated genes and included these genes in our validation gene set (*SLC2A6*, *FAM116B*, and *C17orf56*). Not only were these genes significantly associated with frailty in the European ancestry, elderly, frail in the Vitality study, they were also significantly associated with frailty in our cohort of middle-aged blacks and whites, suggesting that these genes may have important roles in frailty regardless of age or race. El Assar et al. [22] examined gene expression in 350 individuals of European ancestry, non-frail and frail, who were 65 years old and older within the Toledo Study of Healthy Aging, a part of the Frailomic Initiative [22]. In this study, the expression of 21 genes associated with aging or the response to oxidative stress were examined in the context of frailty. We also examined expression of these genes in our cohort and identified significant differential expression within our RNA sequencing cohort of four genes of the 21 genes reported by the Toledo Study investigators, *TXNRD1*, *HMOX2*, *PTGS2*, and *EGLN3*. However, we did not further examine gene expression of *TXNRD1, HMOX2*, and *EGLN3* in the validation cohort because the log2 fold change was less than 1, thus not meeting our selection criteria. However, *PTGS2* was one of the putative frailty-associated genes selected in the Toledo Study and met our criteria for validation. However, there were no differences in *PTGS2* levels across frailty and race groups in our cohort. The failure to replicate the Toledo findings may be related to differences in the characteristics between the 2 cohorts. The Toledo Study Cohort is substantially older with a mean age of 76.5 years for the overall cohort and 83.0 years for frail participants. The Toledo cohort is a non-diverse European ancestry group and importantly only 22% of the 350 participants are frail. The HANDLS cohort is approximately 36 years younger, consists of blacks and whites, and importantly is balanced for percentage of frail individuals. Another group examined gene expression in robust, pre-frail and frail individuals in the Singapore Longitudinal Study of Aging in response to influenza vaccination [23]. Microarray of peripheral blood mononuclear cells prior to vaccination showed no gene expression differences between the robust, pre-frail or frail participants. Influenza vaccination altered gene expression, however, there were no significant differences between groups that exceeded two-fold differential expression.

Race influenced the transcriptional profiles in our cohort. In our RNA sequencing analysis and cohort, we observed significant gene expression differences between non-frail and frail blacks and whites. There were a larger number of differentially expressed genes associated with frailty in whites when compared to blacks (Figure 2B). The most robust differences were in genes that were more abundant in frail, white individuals compared to non-frail individuals. Many of these genes are significantly more abundant only in whites and not in blacks. The parametric analysis of gene set enrichment (PAGE) and network analyses in the context of frailty showed that the enriched biological processes in pre-frail/frail, middle-aged blacks are associated with immune response, inflammatory response, and chemotaxis (Figure 3), which is consistent with the idea that frailty is a chronic state of inflammation that adversely impacts several organ systems [17]. Interestingly, the opposite pattern was observed in whites in that biological pathways associated with immune response, inflammatory response, and chemotaxis were diminished in pre-frail/frail middle-aged whites (Figure 4) which is consistent with racial differences associated with inflammation-related processes [24]. Enriched biological processes in pre-frail/frail whites are sensory perception of taste, tRNA processing, and sensory detection of bitter taste (Figure 4). Although decreased smell and taste perception is associated with frailty in an elderly Japanese population [25], our data suggest that these processes are also impacted in middle-aged, frail whites. Collectively, these data suggest that there are differing molecular signatures for frailty between blacks and whites. This may aid in identifying the underlying differences that may explain the frailty prevalence differences between blacks and whites under the age of 50 [10].

Our RT-qPCR validation study in an expanded cohort of non-frail and frail individuals focused on the 36 genes that we found were significantly changed with frailty status, race or race by frailty in our cohort, and that we found by reanalysis of data from the Vitality 90+ study [18]. The three genes we validated from the Vitality study were *SLC2A6*, *FAM116B*, and *C17orf56. SLC2A6* encodes for a recently identified solute carrier family 2 facilitated glucose transporter member 6 and is highly expressed in lymphocytes; however, the physiological substrate has yet to be identified [26]. SLC2A6 is a member of class III glucose transporters and possesses dileucine motif that facilitates its translocation to organelle membranes. This suggests that SLC2A6 could facilitate glucose transport across intracellular organelles and warrants further investigation in the context of frailty. Increased *SLC2A6* levels were found in frail nonagenarians [18], but in our validation cohort, we found lower levels of *SLC2A6* in frail, middle-aged individuals. These differences could be attributed to racial homogeneity and advanced age of the Vitality cohort in contrast to ours.

Decreased levels of *FCGR3B* were associated with frail individuals in our validation cohort. *FCGR3B* encodes for a membrane bound receptor that is important for calcium mobilization and neutrophil degranulation. Dysregulation of calcium homeostasis is a hallmark of aging and aging-related diseases [16] while dysregulated neutrophil degranulation is a feature of several inflammatory diseases [27]. Therefore, it is interesting to hypothesize that lower levels of FCGR3B may contribute to frailty through dysregulation of calcium homeostasis and through inflammation, both well-established contributors to the frailty phenotype.

*C17orf56* encodes for an accessory protein that is a part of a protein complex that plays an important role in endocytosis and clathrin and non-clathrin vesicle formation [28,29]. Here, we found that *C17orf56* levels were lower in frail individuals which is contrary to what was observed with frailty in the Vitality 90+ nonagenarian cohort. A hallmark of aging is loss of proteostasis [30]. Maintaining protein homeostasis is mediated, in part by endocytosis [31], making it interesting to speculate that dysregulation of proteostsis may occur in younger frail individuals. Future work lies in experimentally testing this idea.

In addition to validating gene expression patterns of frailty-associated genes identified in the Vitality 90+ and Toledo studies, we selected genes (*OSM*, *CXCL1*, and *IL6)* that encode cytokines that were found in biological pathways we identified in our PAGE analysis (Table 2). Oncostatin M (*OSM*) is a diverse cytokine that has recently been identified as a member of the interleukin 6 (IL6) subfamily and also stimulates IL6 production [32]. The chemokine (C-X-C motif) ligand 1 (*CXCL1*) has a role in inflammation and neutrophil recruitment [33]. Elevated levels of IL6, a proinflammatory cytokine [34], have been detected in old, frail cohorts and have been considered a potential frailty biomarker [35]. Interestingly, increased expression of *OSM, CXCL1*, *IL6* were not associated with frailty in our cohort. However, race was the driver for increased expression of all three genes in whites when compared to blacks. These data suggest that previously identified markers of frailty may not be appropriate markers for frailty in middle-aged adults or among blacks cohorts.

We also observed interactions between race, frail status, and expression of interleukin 1 beta (*IL1B)* and the early growth response 1 *(EGR1*) genes. We observed that frail whites have lower levels of both *IL1B* and *EGR1* compared to non-frail whites. Interestingly, this observation was only in whites, as expression of *IL1B* and *EGR1* are increased with frailty in blacks. *IL1B* is an important mediator of the inflammatory response and fever induction [36]. *EGR1*, however, has been investigated in the central nervous system as it is a transcription factor that is required for differentiation, associated with neuroplasticity [37], and synaptic exocytosis [38]. Although neither gene has been previously studied in frailty, and the functional role of EGR1 has not been extensively investigated in peripheral blood cells, the results from this study indicate that these genes may be novel biomarkers of frailty in younger cohorts.

This current study builds upon our understanding of frailty at the molecular level as we examined the transcriptome of middle-aged blacks and whites. Interestingly, the results from the RNA sequencing, PAGE analysis, and interaction network analysis indicate that race is a driver for the changes that we see in the transcriptional profile of frail, middle-aged adults. This is important considering that we found previously in HANDLS that frailty prevalence in adults under 50 is greater in whites than blacks [10].

Our study is limited by the sample size, however, similar sample sizes have previously been reported [39–42]. The strengths of the study are the use of next generation sequencing combined with pathway analysis and RT-qPCR validation in a diverse, community-based cohort.

Frailty is a risk factor for disability and mortality. Previous clinical and epidemiologic work in frailty, ours and others, highlights the need to clinically identify frailty earlier in the lifespan. The current work suggests that it is imperative to study the molecular aspects of frailty prior to old age to enable the identification of early molecular drivers of frailty that may aid in intervention to prevent adverse outcomes.

## METHODS

### Study participants and frailty assessment

Participants are from the Healthy Aging in Neighborhoods of Diversity across the Life Span (HANDLS) study of the National Institute on Aging Intramural Research Program (NIA IRP), National Institutes of Health. HANDLS is an epidemiologic, longitudinal study in which age-related health disparities are examined in the context of behavioral, psychosocial, and environmental influences [43]. The HANDLS cohort includes 3720 white and black residents of Baltimore, Maryland between the ages of 30-64 at baseline. HANDLS has been approved by the Institutional Review Board of the National Institute on Environmental Health Sciences and all participants provided written, informed consent. Participants within the RNA sequencing (n = 16) and validation cohorts (n = 52; 14 overlap with the RNA sequencing cohort) were stratified across sex, race, frailty status, and were between 45-49 years old (Table 1 A and B). FRAIL scores for the different domains were the same for blacks and whites (Supplementary Table 7). Additionally, when stratifying based on the types of illnesses within the illness domain, there were no differences between blacks and whites (Table 3).

**Table 3:** Comorbid illnesses in frail blacks and whites in the validation cohort.

	Blacks	Whites	p value
n	13	13	
Diabetes = Yes (%)	7 (53.8)	2 (15.4)	0.10
Hypertension = Yes (%)	10 (76.9)	7 (53.8)	0.41
Myocardial Infarction = Yes (%)*	0 (100.0)	0 (100.0)	1
Congestive Heart Failure = Yes (%)*	0 (100.0)	0 (100.0)	1
Angina = Yes (%)*	2 (16.7)	2 (16.7)	1
Asthma = Yes (%)*	2 (16.7)	4 (33.3)	0.64
Stroke = Yes (%)*	0 (0.0)	0 (0.0)	1
Kidney Disease = Yes (%)*	0 (0.0)	2 (16.7)	0.46
Cancer = Yes (%)*	0 (0.0)	2 (16.7)	0.46
Chronic Lung Disease = Yes (%)*	2 (16.7)	4 (33.3)	0.64
Arthritis= Yes (%)*	5 (41.7)	4 (33.3)	1
Chronic Diseases (mean (sd))*	2.17 (1.75)	2.25 (1.60)	0.90

*Information for these illnesses were missing from one black and one white individual. Fisher’s exact test was used to analyze differences for categorical variables and Student’s t-test was used for the continuous variable.

Frailty was assessed previously [10] using the FRAIL scale [9] with adaptions for the loss of weight domain [10,44]. We assessed loss of weight from responses to item two of the Center for Epidemiologic Studies Depression scale (CES-D) [45]. Weight loss was considered present when participants responded occasionally (3–4 days a week) or mostly (5–7 days a week) to the following question: Over the past week did you not feel like eating or have a poor appetite?. The International Academy on Nutrition and Aging FRAIL scale consists of five domains: fatigue, resistance, ambulation, illnesses, and loss of weight. The frail score ranges from 0 – 5, in which one point can be received for each component. A score of 0 is non-frail, 1-2 is pre-frail, and 3-5 is frail. In the RNA sequencing cohort, 8 participants were non-frail, 2 were pre-frail, and 6 were frail.

### Next generation sequencing

Peripheral blood mononuclear cells (PBMCs) were isolated from fasting blood samples and stored at -80^o^C [46]. Total RNA was isolated using TRIzol® (Life Technologies) according to the manufacturer’s instructions with the addition of a DNase treatment and overnight precipitation. Samples were resuspended in 20 µl RNase-free water and frozen at -80°C until further use. Next generation RNA-seq was performed at the Johns Hopkins University Deep Sequencing and Microarray Core Facility. Libraries were prepared with the TruSeq stranded total RNA with Ribo-Zero Gold kit (RS-122-2301). The libraries were multiplexed into 4 plex per pool and sequenced on Illumina HiSeq 2500 and the associated Illumina RTA software (Version number 1.18.66) was used to process the images and generate base-calls to perform the primary analysis. The FASTQ file was then used for sequence input for alignment to the human genome NCBI build v38 using TopHat version 2.21 (Figure 1B). Transcripts were assembled using Cufflinks version 2.2.1, Cuffmerge version 2.2.1 and differential gene expression was determined using Cuffdiff version 2.2.1. Significance was determined using the q-value (FDR adjusted p-value using Benjamini-Hochberg correction for multiple-testing) less than 0.05 and also log2 fold change > 0.58 or < -0.58. RNA-seq data can be accessed at GEO (Accession number: GSE129534). All genes significantly associated with frailty are listed in Supplementary Table 1, Supplementary Table 2 and Supplementary Table 3 list genes significantly altered by frailty in blacks and whites, respectively.

Quantile normalized FPKM values were used to calculate Z-scores (log transformed). Z-ratios were then obtained and imputed into PAGE analysis to analyze gene ontology (GO) terms (Figure 1B) [47]. Significance was based on a false discovery rate of < 0.30, p < 0.05, and at least three genes for that significant gene set. In order to visualize functional interactions between significant, differentially expressed genes, we used the Search Tool for the Retrieval of Interacting Genes (STRING) [21,48]. The interactions were analyzed according to the highest confidence score (0.900) clustered using k-means clustering and disconnected nodes were removed.

### Reverse transcription and real-time, quantitative PCR

Total RNA was isolated from PBMCs as described above. 500 µg of total RNA was reverse transcribed into cDNA using random hexamers and Superscript II Reverse Transcriptase (Invitrogen). Reactions were performed with 2X SYBR Green Master Mix and gene-specific primers (Supplement Table 6). Reverse transcription and quantitative real- time PCR (RT-qPCR) reactions were performed on a 7900HT Fast Real-Time PCR System according to the manufacturer’s protocol. mRNA levels were normalized to the average of *GAPDH* and *ACTB*. Gene expression was calculated using the 2^−ΔΔCt^ method [49].

### IL6 and IL1β enzyme-linked immunosorbent assays

IL6 and IL1β levels were measured in serum following the manufacturer’s guidelines (Quantikine ELISA Human IL1B/IL-1F2 and Human IL-6; R&D systems). Optical density was measured using the SpectraMax M2 system (Molecular Devices) along with SoftMax Pro (Version 7). IL6 and IL1β concentrations were calculated according to the respective standards. Log transformed values were used to examine the correlation between serum and PBMC mRNA levels using Pearson’s correlation.

### Statistical approach

The balanced design across sex, race and frailty status for the validation cohort provided 84% power to detect differences in relative expression that accounted for 15% of the total variance. Linear regression models were used to examine the relationship of mRNA levels with frailty status and race. Models were built using forward and backward selection to assess possible interactions among the variables. Log transformations (base 2) were applied for non-normally distributed variables. The association between frailty domains and race were determined using Fisher’s exact tests. Analyses were performed using R [50]. A p-value less than 0.05 was considered to be statistically significant.

## Supplementary Material

Supplementary Table 1

Supplementary Table 2

Supplementary Table 3

Supplementary Table 4

Supplementary Table 5

Supplementary Table 6

Supplementary Table 7
